# Transient effect of weak electromagnetic fields on calcium ion concentration in *Arabidopsis thaliana*

**DOI:** 10.1186/1471-2229-9-47

**Published:** 2009-04-30

**Authors:** Alexander Pazur, Valentina Rassadina

**Affiliations:** 1 Department Biology I (Botany), Ludwig Maximilians University Munich, Menzinger Str. 67, D-80638 Munich, Germany; 2 Institute of Biophysics and Cell Engineering, National Academy of Sciences of Belarus, Academicheskaya 27, Minsk 220072, Belarus

## Abstract

**Background:**

Weak magnetic and electromagnetic fields can influence physiological processes in animals, plants and microorganisms, but the underlying way of perception is poorly understood. The ion cyclotron resonance is one of the discussed mechanisms, predicting biological effects for definite frequencies and intensities of electromagnetic fields possibly by affecting the physiological availability of small ions. Above all an influence on Calcium, which is crucial for many life processes, is in the focus of interest. We show that in *Arabidopsis thaliana*, changes in Ca^2+^-concentrations can be induced by combinations of magnetic and electromagnetic fields that match Ca^2+^-ion cyclotron resonance conditions.

**Results:**

An aequorin expressing *Arabidopsis thaliana *mutant (*Col0-1 Aeq Cy+*) was subjected to a magnetic field around 65 microtesla (0.65 Gauss) and an electromagnetic field with the corresponding Ca^2+ ^cyclotron frequency of 50 Hz. The resulting changes in free Ca^2+ ^were monitored by aequorin bioluminescence, using a high sensitive photomultiplier unit. The experiments were referenced by the additional use of wild type plants. Transient increases of cytosolic Ca^2+ ^were observed both after switching the electromagnetic field on and off, with the latter effect decreasing with increasing duration of the electromagnetic impact. Compared with this the uninfluenced long-term loss of bioluminescence activity without any exogenic impact was negligible. The magnetic field effect rapidly decreased if ion cyclotron resonance conditions were mismatched by varying the magnetic fieldstrength, also a dependence on the amplitude of the electromagnetic component was seen.

**Conclusion:**

Considering the various functions of Ca^2+ ^as a second messenger in plants, this mechanism may be relevant for perception of these combined fields. The applicability of recently hypothesized mechanisms for the ion cyclotron resonance effect in biological systems is discussed considering it's operating at magnetic field strengths weak enough, to occur occasionally in our all day environment.

## Background

Effects of weak static magnetic (MF) and electromagnetic fields (EMF) on plants were investigated since more then three decades, even though the number of studies is small compared to those performed on animals and humans [1]. Under the aspects of ecology and environmental sciences two influences are here in the focus of interest: Firstly the ubiquitous geomagnetic field with its location-, direction- and time-dependent variations in the range from 30–70 μT, and low frequency EMF natural sources given by electromagnetic processes in the atmosphere [2,3] and secondly, man made sources like electric power lines and wireless communication. Commonly 3 types of magnetoreception are discussed in biology: ferrimagnetism, electron spin controlled chemical reactions by radical pairs, and the magnetic forcing on small ions.

Ferrimagnetic particles were related in several animals to magnetic field perception [4]. They were also found in plants, e.g. a *Festuca *species [5], but their size and concentration appear too low for generating a sufficient magnetic force. The radical pair effect [6] requires a transient formation and recombination of radical pairs. Recombination can result in either singlet or triplet states, with the relative ratios, and thereby also that of subsequent products, being affected by weak magnetic fields. The mechanism has been studied in detail *in vitro*, e.g. in photosynthetic systems, but recently cryptochrome-dependent responses were investigated *in vivo*, e.g. in *Arabidopsis *[7,8].

Search for other mechanisms was triggered by the finding that MF and EMF effects could be observed with many organisms without proven ferrimagnetic particles, and at field strengths well below those required for the radical pair mechanism (see [9] for leading references). An indication to such a mechanism arose when "windows" of optimal effectiveness were seen for certain combinations of field strengths and frequencies of the applied MF and EMF [10]. A superposition of the static and the alternating field component was needed to match such an "effectiveness window", with a definite frequency *f*, and an amplitude **B**_***AC ***_commonly weaker than the flux density **B**_***DC ***_of the applied MF. This non-linear dose-response effect was first related by Liboff to ion cyclotron resonance (ICR) of small ions [11]. The MF and EMF components were related to the equation for the cyclotronic frequency *f *of charged particles in a MF,

(1)

where mass *m*_*i *_as well charge *Q*_*i *_corresponded to one of the small ions in the electrolytes of the test object. This mechanism could be verified in several animal, plant and microorganism species [12-14]. It was clearly demonstrated that a definite effect can be produced by tuning to the ICR fundamental frequencies for physiologically important cations like Ca^2+^, Mg^2+ ^or Na^+^. Changes in plant development and morphology were observed after breeding in MF+EMF parameterized to the Ca^2+^-ICR condition. Radish (*R. sativus*) showed slowed germination, but stimulated growth after exposure to Ca^2+^-ICR conditions [15]. Under similar conditions, germinating beans showed increased radicle lengths, which additionally depended on the external Ca^2+ ^concentration [13]. Barley plants had deficiencies in growth, water uptake and photosynthetic pigment synthesis that pertained for several weeks after a treatment during the first 5 days of germination with field frequency combinations matching a Ca^2+^-ICR condition [16].

Ca^2+ ^regulates diverse cellular processes in plants as a ubiquitous internal second messenger, conveying signals received at the cell surface to the inside of the cell through spatial and temporal concentration changes that are decoded by an array of Ca^2+ ^sensors [17-20]. Elevated concentrations of cytosolic free calcium ([Ca^2+^]_cyt_) are induced in response to various stimuli, such as red light, mechanic stimulation, cold shock, gravity, pathogen attack, and phytohormones [19,21,22](see also references therein), further by drought and soil salinity [23]. During these processes, [Ca^2+^]_cyt _levels rise via gated Ca^2+ ^channels that are located on the plasma membrane and intracellular membranes. The next stage in transmitting the Ca^2+ ^signal within the cell is related to the signal "decay"; it represents the active removal of excess Ca^2+ ^from the cytosol to the extracellular medium or organelles by means of Ca^2+^-ATPases and/or Ca^2+^/H+ antiporters. The primary intracellular targets of Ca^2+ ^are various Ca^2+^-binding proteins; they ensure Ca^2+ ^transport, serve as a Ca^2+ ^buffer, or translate the Ca^2+ ^signal to intracellular signal chains and initiate Ca^2+^-dependent physiological processes.

In our previous long term study [16], we provided indirect evidence for the impact of MF+EMF parameterized to the Ca^2+^-ICR condition, on processes of plant development largely regulated by this ion. We now show that in a bioluminescent aequorin-mutant of *Arabidopsis thaliana *[24] changes in free Ca^2+ ^could be directly monitored when field combinations were applied that match ICR conditions for Ca^2+^, and that these effects fall off when the conditions were detuned, or the intensity of the electromagnetic field was reduced.

## Methods

### Plant materials and growth conditions

The aequorin producing mutant *Col0-1 Aeq Cy+ *of *Arabidopsis thaliana *(AEQ) was a kind gift of P. Galland (University of Marburg). It is a stem of biotype background Columbia and the cytosolic apoaequorin expression is controlled by the cauliflower mosaic virus promoter 35S [25]. The *Arabidopsis thaliana *wild type used for control experiments was taken from an in-house stock (Ecotype *Col-0*). Both types of seeds were cultivated according to Plieth and Trewavas [24], with the following exceptions: Seeds were disinfected first with 70% ethanol (2 min) and then with a 5% aqueous solution of "DanKlorix" cleaner (Colgate-Palmolive, Hamburg) (15 min), and washed thoroughly 5 times with distilled water.

Sterile agar plates containing 1.2% agarose (Merck, 1.07881) without additional sucrose were performed and stocked up in a refrigerator at +4°C, and warmed up to room temperature directly before use. Seeds were placed manually using an inoculation loop on the agar plates on a laminar flow hood, stored at 4°C for 48 h for vernalization, then incubated for 24 h under white fluorescent light (4600 lux), and finally kept in the dark for 4 days, at 21 ± 0.2°C. Thereafter the plants were grown at the same place with a 12 h light (4600 lux)/12 h dark period. After 10–12 days germinated plants had a more or less uniform shoot size of 5–7 mm and grew with an average distance of 1–1.5 cm on the agar, which facilitated later measuring on single plants by using a mask of black cardboard above the petri dish for selecting individuals.

On the day before measurement the cytosolic aequorin was reconstituted. An aliquot (42.5 μL) of a stock solution of coelenterazine (1 mg, 07372-1MG-F, Sigma-Aldrich Germany) in ethanol (1 ml) was diluted with doubly distilled water (10 ml). The agar plates of the AEQ as well as the wild type plants were completely covered with this solution about 1 mm and incubated for 6 h in the dark. That warranted, that coelenterazine was available sufficiently, independent from the respective number of plants. Afterwards the supernatant liquid was removed, and the plates stored overnight in a dark box in the measuring room in order to minimize temperature- and mechanical stress of transportation before the measurements. All procedures with the Petri dishes opened were performed on the laminar flow hood. Subsequently there was no need for opening the Petri dishes for the optical measurements itself.

### Magnetic field experiments

**Figure 1:**
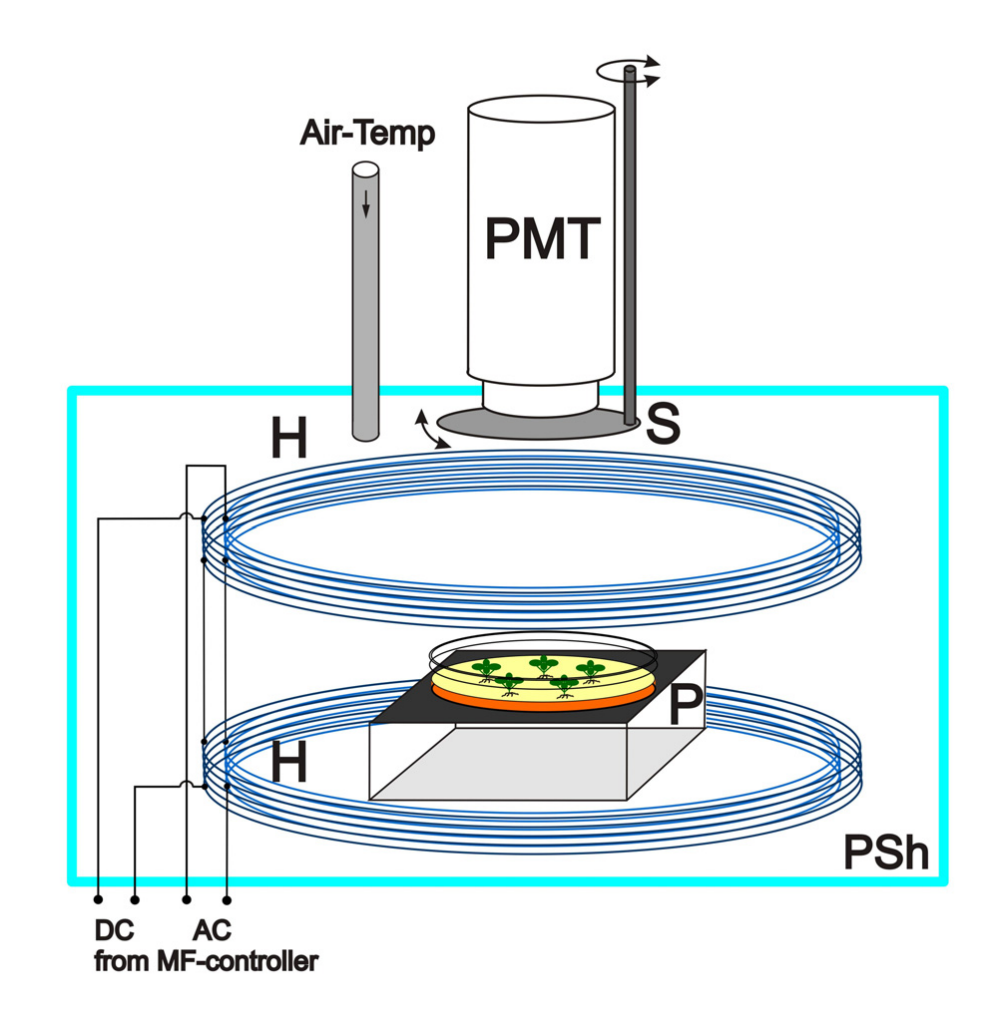
**Experimental setup (schematic) for monitoring cytosolic Ca^2+ ^by aequorin bioluminescence in a combination of magnetic (MF) and electromagnetic (EMF) fields**. The Petri dish containing the sample seedlings (P) is positioned axially in the center of two pairs of Helmholtz coils wired one upon the other (H) and connected to control units, which generate the static and modulated magnetic fields. Coils and sample are magnetically shielded by a permalloy housing (PSh), that reduces any external MF and EMF to some percent in comparison to the fieldstrength applied inside, reversely even least magnetic retroactions to the components outside can be avoided. Aequorin bioluminescence was detected by a vertically mounted photomultiplier tube, which can be closed manually by a shutter (S). The temperature was adjusted by directing a gentle flow of temperature stabilized air into the sample compartment.

The bioluminescence of aequorin was measured in a modular spectrofluorimeter (Spex Fluorolog 1), a similar instrumentation was described by Carson and Prato [26]. The samples were placed in a permalloy shielding box (metal sheets 1 mm thick) that contained two pairs of Helmholtz-coils (inner diameter 13 cm), wired one on to the other, with 200 and 100 turns for the DC and AC magnetic field generation, respectively (Fig. 1). The first coil pair was connected to an adjustable DC power supply with an accuracy of 0.2% and a noise factor of <0.1% referring to the coil current. The second coil pair was driven by a function generator producing a 50 Hz sinusoidal signal, which was phase-locked with the power frequency. Thus almost any residual noise from surrounding electric facilities (50 Hz and its overtones) could be eliminated by a degeneration circuit, and interference was avoided. This technique was successfully used earlier by Pazur et al. [16] and allows a controlled application of this important civilizing EMF frequency.

At this frequency, ICR conditions for Ca^2+ ^are matched at B_DC _= 65.8 μT (eq. 1). The sample dish was placed in the center of the vertical axis of the coil pairs, where a homogeneity error of the field <3% could be reached across the area of optical detection of about 20 cm^2^. The MF field strength and EMF amplitude were monitored by a fluxgate teslameter FM GEO-X (Projekt Elektronik GmbH, Berlin) directly underneath the sample. Intensity and timing of MF and EMF were controlled by a personal computer with a 12-bit DA-converter board. For reaching a constant temperature of 21 ± 0.5°C, a slight temperature stabilized airflow (20–22°C, dependent from the room temperature) of about 0.5 l/min was guided into the chamber, and the temperature monitored by a digital thermometer.

The temperature equilibrated Petri dishes were inserted in the measurement chamber. The lid was closed and, as a precaution, additionally covered by a black cloth. 30 min after switching on the high voltage of the photomultiplier tube, the system seemed to have reached a stable operating point, and the initially increased AEQ luminescence, possibly caused by the prior handling of the plants, had decreased to a constant level. The bioluminescence was detected by a front-end photomultiplier Type R374E (Hamamatsu) operated at a cathode voltage of -1000 V, which had a high quantum efficiency at 400–500 nm wavelength. It was mounted axially in a shielding tube with a face to face distance of 7.5 cm to the sample dish and an aperture angle of 30°. A rotating sheet of black plastic served as a shutter (Fig. 1). The signal of the photomultiplier was digitized by a 12-bit AD-Converter, and fed into a personal computer using a home-made software. Shown data are averages of at least 5, these for B_DC _= 65.8 μT, B_AC _= 5 μT of 13 individual experiments with separate plant cultures. The course of the luminescence intensity could be monitored for extended periods (>2 h) of time with a resolution of 6 s. Data from 5–13 independent experiments for each of 9 categories were normalized and analyzed using Microsoft^® ^Excel. Additionally the photon flux could be calculated using the manufacturer data sheet for the photomultiplier, a Gauss distributed spectral band with a maximum around 465 nm with a peak width at half-height of 80 nm was assumed therefore [27]. The emission spectrum of bioluminescence itself could not be analyzed experimentally in default of a suitable monochromator.

## Results

The germination rate of the AEQ seeds after 10 days was significantly lower (38 ± 7%) than that of the wild type (92 ± 5%). The effectiveness of the AEQ gene expression in the mutants layed at 45 ± 7% in 5 tests with 85 plants in total. That came up to the expectation, because the AEQ plants were heterozygous. It was discernible by the enhanced steady state bioluminescence from single plants, which could be optically selected by a relocatable cardboard. For 10 days old AEQ seedlings it was about 3–5 times above the dark signal and corresponded to about 2.6·10^4 ^photons/cm^2^·s by the assumptions described above, inspecting simultaneously 10–12 plants in the most cases. The usable full scale range of the detection system would amount to 5.3·10^8 ^photons/cm^2^·s by this scale. The absolute level of bioluminescence depended from the respective number of seeds per plate, size, and the coelenterazine uptake of the plants. Wild type plants showed no signal above the dark level after incubation with coelenterazine. Because the photomultiplier unit was outside the permalloy shielding box with the coils, an influence of the relatively weak MF on the photomultiplier could be excluded, but was nevertheless checked for safeness, as well in the total dark as with a piece of a phosphorescing clock face as a low light source. There was still no effect at 5 mT, the available maximum intensity of the apparatus, which was the about hundredfold of that used for the experiments.

### Response to MF/EMF combinations matching Ca^2+^-ICR

**Figure 2:**
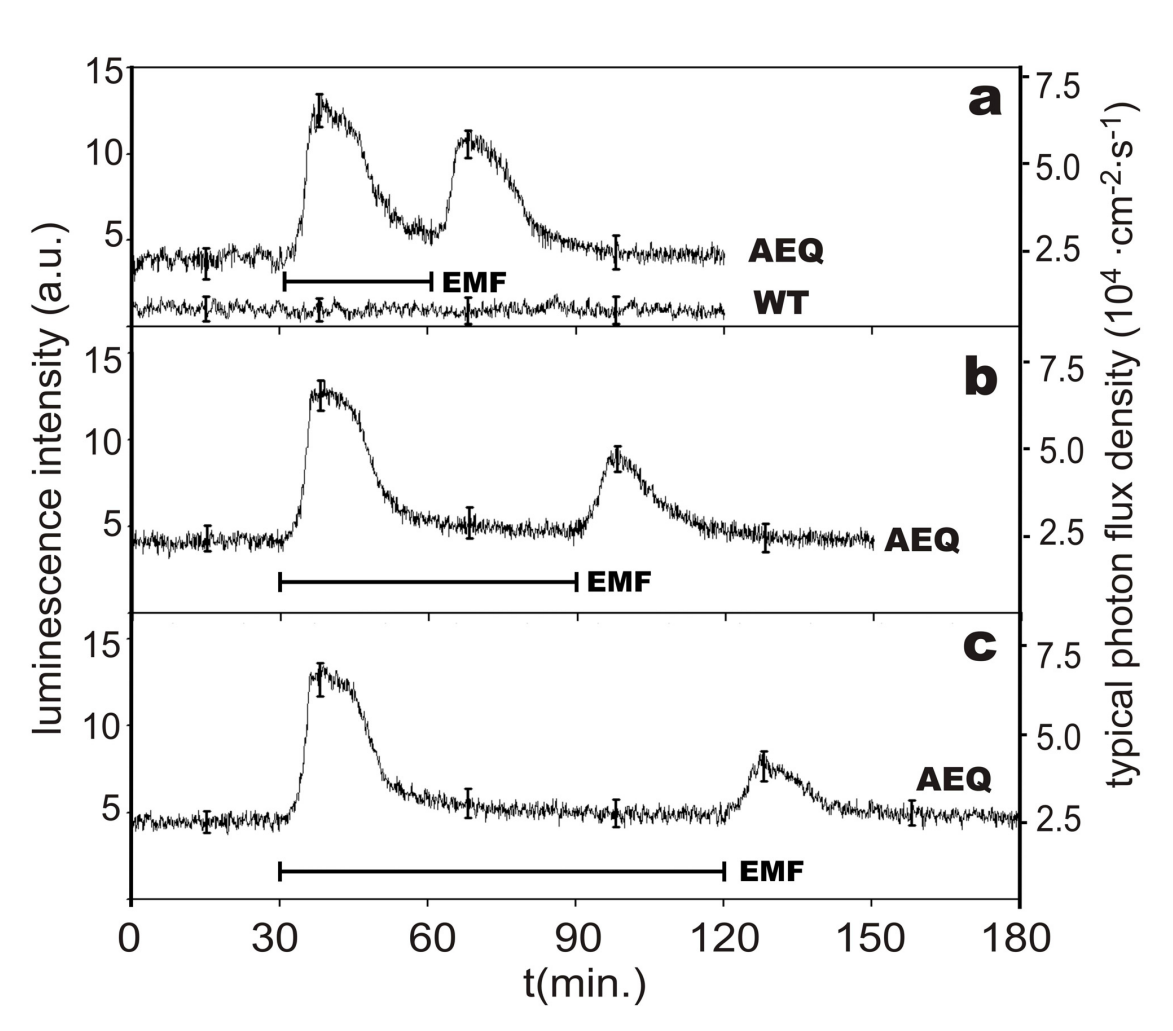
**Bioluminescence response of the *Arabidopsis *aequorin mutant (*Col0-1 Aeq Cy+*) to a combined MF (B_DC _= 65 μT) and EMF (*f *= 50 Hz, B_AC _= 5 μT), matching Ca^2+^-ICR conditions (a-c)**. The horizontal bars below the graphs indicate the time of Ca^2+ ^– ICR condition. All data are normalized against the dark current signal, which also corresponds to control experiments with wild-type plants (labelled as WT). The vertical bars on the curves mark the standard deviation at the indicated positions.

A static MF for the desired condition was applied continuously to the seedlings during the whole experiment. According to eq.(1) it was related to an additional 50 Hz EMF, running without any interference to the power frequency like described above. Before enabling the ICR condition by applying the EMF, the photocurrent was monitored for 30 min to ensure a stable background. After switching on the EMF, the bioluminescence of the AEQ plants increased significantly. After an initial lag-phase of 20–30 s, it rose within 7–8 min to a maximum that was about 3-fold higher than the basic level before EMF application. Subsequently, the signal intensity decreased again, and relaxed to nearly the original value after about 30 min (Fig. 2). This indicates a transient increase of the free cellular Ca^2+ ^concentration that is induced by the EMF. In 8 independent experiments with different cultures, the maximum of the EMF-induced transient was 3.1 ± 0.26 times above the basal level. A second transient increase in [Ca^2+^]-stimulus was obtained when the EMF was switched off. It had similar kinetics, but only 2/3 the intensity of the "on-peak". 30 min after the "off" stimulus the aequorin-luminescence has been largely relaxed, but complete return to the basal level needed at least 60 min (Fig. 2b, c). Both the transients after turning the EMF on and off were well reproducible, the experiments shown in Fig. 2 were averages of 13 and 10 experiments, respectively. With increasing duration, the "off" response became weaker. This could be related to the interval between the two, or more precisely to the time the EMF was applied. One possible reason for the fading effect could be due to the progressive consumption of available coelenterazine, but the hourly long term loss of bioluminescence capability lay at only 3.4%, which corresponded to a half-life period of about 22 h. Hence a spatial, redistribution of cytoplasmatic Ca^2+^, finally more inconvenient for the ICR effect, could also be responsible. No transients were seen in any of the control experiments with wild-type seedlings under identical conditions.

### Detuning from Ca^2+ ^resonance conditions

**Figure 3:**
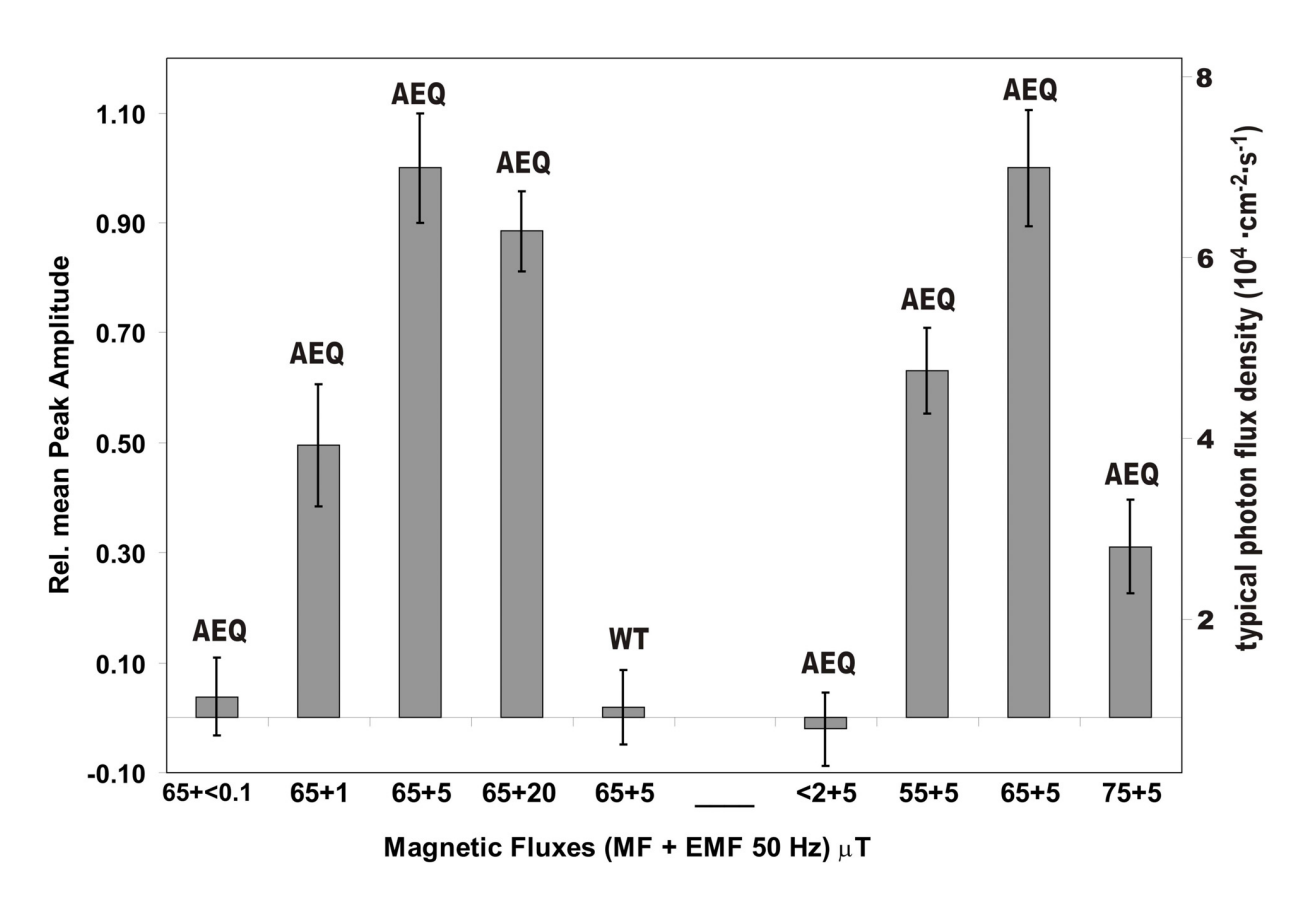
**Response of bioluminescence in *Arabidopsis *aequorin mutant (*Col0-1 Aeq Cy+*) and wild type plants to different combinations of static and modulated (50 Hz) MF/EMF**. These were achieved by varying the fieldstrength around the optimized ICR condition for Ca^2+ ^(**B**_DC _= 65 μT, **B**_AC _= 5 μT, *f *= 50 Hz). The 30 min. integral values are normalized to the corresponding 30 min. integral of the optimized condition. Experimental conditions: EMF of **B**_AC _< 0.1 (background noise), 1, 5, and 20 μT were used with a MF of 65 μT (left 4 bars), and MF of **B**_DC _<2 (background), 55, 65, and 75 μT were combined with an EMF amplitude of 5 μT (right 4 bars). The data for the *Arabidopsis *wild type plants at the optimized condition are labelled as WT. Standard deviations are shown by the vertical bars.

The described experiments were performed such that the MF field strength and EMF frequency matched ICR conditions for Ca^++^. In order to prove that we observe indeed a resonance effect, the ICR conditions were detuned in the following experiments by changing the MF, and the ensuing Ca^++ ^concentrations were again monitored by the aequorin bioluminescence. First, the EMF field strength was varied in order to find an optimum strength for the subsequent experiments with variable MF. The left bars in Fig. 3 present the 30 min. integral of luminescence above the background after switching on an EMF at four different field strengths. An EMF of B_AC _< 0.1 μT showed no effect, as also did the wild type plants used as control at any condition tested. A clear transient Ca^2+^-increase was already observed for an EMF with B_AC _= 1 μT, and saturation was reached at 5 μT; the data are normalized to this level. Setting the EMF to B_AC _= 5 μT, the strength of the static MF was detuned from the ICR condition (eq. 1). Both with a B_DC _lying 10 μT below (55.8 μT) or above (75.8 μT) the Ca^2+^-ICR condition at 65.8 μT, there was a significant decrease of the transient signal (Fig. 2). The right 4 bars (Fig. 3) show the 30 min. integral of luminescence above the background after switching on the EMF; the response is significantly decreased with both lowered and increased B_DC_. We also tested the effect of the residual MF of about 2 μT in the shielding box: there was no measurable change in the luminescence of the AEQ plants after switching on the EMF, indicating that there is no influence to cytosolic Ca^2+^-concentrations at the 50 Hz EMF solely without a MF according for both to an ICR condition (eq. 1).

## Discussion

The aequorin producing Arabidopsis mutant *Col0-1 Aeq Cy+ *facilitates a powerful way to study the cytosolic Ca^2+ ^flux in response to exogenic stressors. The lowered germination rates compared to the wild type of this plant seen here also were observed earlier for the overexpression of cytoplasmatic proteins of the Hsp90 family in Arabidopsis [23], but a generalization of this prior finding in our case for Aequorin would remain speculative, also it could be a property of the batch just used. The subsequent calculation of photon fluxes by the data from an integrating detection system is too vague for a conclusion about the absolute Ca^2+ ^concentration changes in the specimen. There would be need for a single photon counter, which was not available. Independent from all these limitations, the results found here suggest for the first time a direct and rapid influence of the resonant electromagnetic excitation of the cyclotronic frequency of Ca^2+ ^on the concentration of this ion in the cytosol. This change is transient and relaxes within ~60 min, and Ca^2+ ^transients were observed both by switching the Ca^2+^-ICR condition on and off. Plants usually maintain a cytoplasmatic free Ca^2+^-ion concentration of 100–200 nM by ion specific membrane channels and storage proteins or organells like the vacuole; higher Ca^2+^-levels are cytotoxic in the long-term [28,29]. Several external stimuli can trigger a transient increase in intracellular Ca^2+^, which in turn triggers a variety of signal chains. The recovery kinetics depend on many factors and the type of stimulus, they vary from seconds to hours. The signal decay within about 30 min seen in the experiments suggests a rather slow regulation process, it is comparable e.g. to that seen for gravitational stimulation [24]. In this study aequorin bioluminescence of the AEQ mutant was used to monitor changes of Ca^2+ ^concentration; it avoids possible interfering stimuli e.g. by light, when fluorescence methods are used [28,30]. Even though the latter methods e.g. by using chlorpromazine, "Fura" or "Fluo-3" give a substantively better signal [31], we considered the AEQ-mutants favourable due to the lack of potential interference and to maintain high selectivity for the magnetic stimuli.

Earlier investigations of MF and EMF effects on Arabidopsis use significant higher magnetic flux densities up to 400 mT [32] and more, but the MF and EMF intensities used in the recent work are weaker by some orders and furthermore the effect depends on the specific charge (*Q*_*i*_/*m*_*i*_) of ions.

Thereby three questions arise, firstly, if an influence of such weak MF and EMF fields on Calcium signaling in living cells would exist in general, which is probably seen by the findings in this field up to now. Further other important ions should also be affected, which also was shown in some cases [33-35]. Not at least the knowledge about the underlying physical mechanism would be essential.

The space needed for an undisturbed movement of an ion in a MF is governed by the *Larmor *radius (eq. 2), which predetermines the minimally required coherence length λ = 2·**r**_**L **_in terms of quantum mechanics. Due to collisions with thermal moving solvent molecules, an undisturbed free distance λ for an ion circulating with the *Larmor *radius **r**_**L **_and speed **v**

(2)

should not be possible in an aqueous phase. This paradox has been addressed earlier by the suggestion, that ion channels and ion-protein complexes guide the ion orbits [11,36,37] and can maintain the necessary coherence length λ = 2·**r**_**L **_of some 10^-9 ^m free from thermic environmental influence. But the ICR effect could be observed even in aqueous solutions of small molecules like glutamic acid [34,35,38] without any additional biological components, and the need arose for a more universal explanation for the ICR effect [39,40]. The existence of dielectric boundaries is common to any biological or in vitro system probed for MF and EMF effects.

Dielectric boundaries build up an electric double-layer (inner and outer *Helmholtz*-layer), the inner layer produces a potential trap for ions directly above the boundary plane between the two phases, and effects a sharp transition zone for relative dielectric permittivity *ε*_*r*_, refraction number and entropy between the two phases. It influences the adjacent diffuse, outer layer, which generates the measurable zeta potential (ζ). The trapped ions should provide an area with a local electric field **E**(*d*) and relative dielectric permittivity ε_*r*_(*d*), at the distance *d *from the phase boundary. An idealized electromagnetic coupling with an external MF (**B**) may then be described by:

(3)

*ε*_0 _is the electric field constant, μ_0 _the induction constant, μ_*r *_the magnetic permittivity number, and *c*' speed of light at *d*. The free coherence length λ then can be estimated by the De Broglie equation (*h *Planck constant):

(4)

Assuming a typical electric double layer e.g. of a cytoplasm membrane, λ~4.7 nm is obtained for Ca^2+ ^and the MF fieldstrength used in the experiments, which is sufficient for the expected Larmor radii **r**_**L **_of < 2 nm in a plane parallel and close to the dielectric surface. According to the *Born *equation, the shielding energy *w*_*S *_caused by an ion trapped in this "two-dimensional cage" or "quantum wall" will overcome the *k*·*T *energy of the thermic environment:

(5)

Important properties of a resonance effect like ICR are reflected in the line width and amplitude of resonant excitation. Both parameters seem to be wide in our experiments (Fig. 3). This is not uncommon for *in vivo *conditions (see Binhi [9] for leading references). The relation of MF fieldstrength and EMF amplitude **B**_AC_/**B**_DC _was selected in many studies in a range 0.3–2 [13,15,41], meaning a B_AC _up to 100 μT. The finding of an effective B_AC _< 100 nT and vanishing of the ICR effect for EMF amplitudes exceeding some multiples of that value by some laboratories [34] nonetheless could indicate a relatively narrow and sharply defined plane, in which *Larmor *orbits lie. Moreover such weak EMF are nearly ubiquitous, caused by natural and man-made phenomena in the atmosphere, enabling many different ICR conditions in combination with the geomagnetic field, by which influences to our health and ecology could arise, above all, if Ca^2+ ^resonance is affected.

## Conclusion

In summary the work presented here shows in *Arabidopsis thaliana *seedlings transient Ca^2+^-responses to MF/EMF combinations matching ICR conditions for this ion. The effects reported here are averaged for the entire plant; they do neither provide resolution over the different organs nor within individual cells. Future work using e.g. Ca^2+^-responsive fluorescent dyes and confocal microscopy will be needed to show if local effects may be even more pronounced.

## Abbreviations

MF: static magnetic field; EMF: electromagnetic (alternating) field; ICR: Ion cyclotron resonance; AEQ: *Arabidopsis thaliana *mutant *Col0-1 Aeq Cy+*.

## Authors' contributions

The authors carried out the experiments, compiled the background information and drafted the manuscript. All authors read and approved the final manuscript.

## References

[B1] Galland P, Pazur A (2005). Magnetoreception in plants. J Plant Res.

[B2] Bhattacharya AB, Chatterjee MK, Bhattacharya R (1999). Electromagnetic noise due to man-made sources and lightning and the possible biological effects – a review. Ind J Radio Space Phys.

[B3] Olson P, Amit H (2006). Changes in earth's dipole. Naturwissenschaften.

[B4] Wiltschko W, Wiltschko R (2005). Magnetic orientation and magnetoreception in birds and other animals. J Comp Physiol A.

[B5] Gajdardziska-Josifovska M, McClean RG, Schofield MA, Sommer CV, Kean WF (2001). Discovery of nanocrystalline botanical magnetite. Eur J Mineral.

[B6] Adair RK (1997). Hypothetical biophysical mechanisms for the action of weak low frequency electromagnetic fields at the cellular level. Radiat Prot Dosim.

[B7] Ahmad M, Galland P, Ritz T, Wiltschko R, Wiltschko W (2007). Magnetic intensity affects cryptochrome-dependent responses in Arabidopsis thaliana. Planta.

[B8] Solov'yov IA, Chandler DE, Schulten K (2007). Magnetic field effects in Arabidopsis thaliana cryptochrome-1. Biophys J.

[B9] Binhi VN, Binhi VN (2002). Magnetobiology. Interference of Bound ions.

[B10] Blackman CF, Elder JA, Weil CM, Benane SG, Eichinger DC, House DE (1979). Induction of calcium-ion efflux from brain tissue by radio-frequency radiation: effects of modulation frequency and field strength. Radio Science.

[B11] Liboff AR (1985). Cyclotron resonance in membrane transport. NATO ASI Series A.

[B12] Liburdy RP, Callahan DE, Harland J, Dunham E, Sloma TR, Yaswen P (1993). Experimental evidence for 60 Hz magnetic fields operating through the signal transduction cascade. Effects on calcium influx and c-MYC mRNA induction. FEBS Lett.

[B13] Sakhnini L (2007). Influence of Ca2+ in biological stimulating effects of AC magnetic fields on germination of bean seeds. J Magn Magn Mater.

[B14] Pazur A, Schimek C, Galland P (2007). Magnetoreception in microorganisms and fungi. Cent Eur J Biol.

[B15] Smith SD, McLeod BR, Liboff AR (1995). Testing the ion cyclotron resonance theory of electromagnetic field interaction with odd and even harmonic tuning for cations. Bioelectroch Bioener.

[B16] Pazur A, Rassadina V, Dandler J, Zoller J (2006). Growth of etiolated barley plants in weak static and 50 Hz electromagnetic fields tuned to calcium ion cyclotron resonance. Biomagn Res Technol.

[B17] Volotovski ID (1998). Ca2+ and intracellular signalling in plant cells: a role in phytochrome transduction. Membr Cell Biol.

[B18] Reddy ASN (2001). Calcium: Silver bullet in signaling. Plant Science.

[B19] Sanders D, Pelloux J, Brownlee C, Harper JF (2002). Calcium at the crossroads of signaling. Plant Cell.

[B20] Yang T, Poovaiah BW (2003). Calcium/calmodulin-mediated signal network in plants. Trends Plant Sci.

[B21] Knight MR (2002). Signal transduction leading to low-temperature tolerance in Arabidopsis thaliana. Phil Trans R Soc Lond B.

[B22] White PJ, Broadley MR (2003). Calcium in plants. Ann Bot-London.

[B23] Song H, Zhao R, Fan P, Wang X, Chen X, Li Y (2009). Overexpression of AtHsp90.2, AtHsp90.5 and AtHsp90.7 in Arabidopsis thaliana enhances plant sensitivity to salt and drought stresses. Planta.

[B24] Plieth C, Trewavas AJ (2002). Reorientation of seedlings in the earth's gravitational field induces cytosolic calcium transients. Plant Physiol.

[B25] Knight H, Trewavas AJ, Knight MR (1997). Recombinant aequorin methods for measurement of intracellular calcium in plants. Plant Mol Biol Manual.

[B26] Carson JJL, Prato FS (1996). Fluorescence spectrophotometer for the real time detection of cytosolic free calcium from cell suspensions during exposure to extremely low frequency magnetic fields. Rev Sci Instrum.

[B27] Shimomura O, Musicki B, Kishi Y (1988). Semi-synthetic aequorin. An improved tool for the measurement of calcium ion concentration. Biochem J.

[B28] Medvedev SS (2005). Calcium signaling system in plants. Russian J Plant Physiol.

[B29] Plieth C (2005). Calcium: Just Another Regulator in the Machinery of Life. Ann Bot-London.

[B30] Plieth C (2001). Plant calcium signaling and monitoring: Pros and cons and recent experimental approaches. Protoplasma.

[B31] Walczysko P, Wagner E, Albrechtova JTP (2000). Use of co-loaded Fluo-3 and Fura Red fluorescent indicators for studying the cytosolic Ca2+ concentrations distribution in living plant tissue. Cell Calcium.

[B32] Takimoto K, Yaguchi H, Miyakoshi J (2001). Extremely low frequency magnetic fields suppress the reduction of germination rate of Arabidopsis thaliana seeds kept in saturated humidity. Biosci Biotechnol Biochem.

[B33] Fesenko EE, Novikov VV, Kuvichkin VV, Yablokova EV (2000). Effect of aqueous salt solutions treated with weak magnetic fields on the intrinsic fluorescence of bovine serum albumin. isolation from these solutions and partial characterization of the biologically active fluorescing fraction. Biofizika.

[B34] Zhadin MN, Novikov VV, Barnes FS, Pergola NF (1998). Combined action of static and alternating magnetic fields on ionic current in aqueous glutamic acid solution. Bioelectromagnetics.

[B35] Pazur A (2004). Characterisation of weak magnetic field effects in an aqueous glutamic acid solution by nonlinear dielectric spectroscopy and voltammetry. Biomagn Res Technol.

[B36] McLeod BR, Liboff AR, Smith SD (1992). Electromagnetic gating in ion channels. J Theor Biol.

[B37] Binhi VN, Alipov YeD, Belyaev IY (2001). Effect of static magnetic field on E. coli cells and individual rotations of ion-protein complexes. Bioelectromagnetics.

[B38] Giuliani L, Grimaldi S, Lisi A, D'Emilia E, Bobkova N, Zhadin M (2008). Action of combined magnetic fields on aqueous solution of glutamic acid: the further development of investigations. Biomagn Res Technol.

[B39] Del Giudice E, Fleischmann M, Preparata G, Talpo G (2002). On the "unreasonable" effects of ELF magnetic fields upon a system of ions. Bioelectromagnetics.

[B40] Liboff AR, Jenrow KA (2000). Cell Sensitivity to Magnetic Fields. Electro Magnetobiol.

[B41] Ruzic R, Jerman I (1998). Influence of Ca2+ in biological effects of direct and indirect ELF magnetic field stimulation. Electro Magnetobiol.

